# Relative age effects in ice hockey extends to coaching

**DOI:** 10.3389/fspor.2024.1507386

**Published:** 2025-01-10

**Authors:** Simon Grondin, Daniel Fortin-Guichard, Jean Lemoyne, François Trudeau, Joe Baker

**Affiliations:** ^1^ École de psychologie, Université Laval, Québec, QC, Canada; ^2^ Department of Kinesiology & Physical Education, McGill University, Montréal, QC, Canada; ^3^ Faculty of Kinesiology and Physical Education, University of Toronto, Toronto, ON, Canada; ^4^ Département des sciences de l'activité physique, Université du Québec à Trois-Rivières, Trois-Rivières, QC, Canada

**Keywords:** relative age effect, coaching, sport, ice hockey, birthdate effect

## Abstract

Date of birth influences the chances of success in sports. Compared to players born just before a cutoff date for marking the admissibility in a category (age groups), players born soon after are overrepresented. However, it is not yet known whether the effect of date of birth in sports applies beyond the players' active participation in the game. The aim of the study was to determine whether there is a date of birth effect among ice hockey's coaches. The birthdates of 3,380 coaches in minor league hockey for the 2023–2024 season were obtained from Hockey Québec. The investigation indicated that people born in the first half of the year were more likely to become minor hockey coaches than those born in the second half. This significant birthdate effect is observed with coaches born in 1980s (53.42% vs. 46.58%) and after 1989 (55.73% vs. 44.27%), but not with coaches born before 1980 where the effect tended to reverse. This finding is interpreted as a consequence of the birthdate effect during the development as a hockey player and suggests a loss of potential coaches to the ice hockey system.

## Introduction

Many studies have shown that the time of birth in the year is a determining factor in sport participation and success among children and adolescents [for reviews, see (1, 2)]. This influence is caused by the fact that groupings into categories in amateur sport are based on chronological age. Children born just after the eligibility date are the oldest in the cohort (i.e., in the age category). Compared to younger children who evolve in the same cohort, they are more likely to have greater physical (height, weight, strength, speed) and psychological (affective and cognitive) maturity, and more experience in the game (2). Consequently, relatively older children can appear to be “better” or more talented at a given sport compared to their relatively younger peers who evolve in the same cohort, which affects their likelihood of being selected in more competitive teams, getting more exposure, which improve their chance for future success such as being selected to competitive teams and/or drafted for upper echelons of competition (3). For example, when January 1st is used as the cutoff date, elite athletes in a given sport are more likely to be born in the first than in the last months of the year (1, 2).

Ice hockey was the first sport for which the impact of birthdate on sport participation was measured (4, 5). While there have been additional reports since exploring Canadian hockey and the National Hockey League (NHL), where most players historically were Canadian [see (6–12)], the problem of grouping players according to age categories in ice hockey extends to other countries (13–15).

Importantly, the issues created by grouping into categories based on chronological age are not restricted to ice hockey. There is an abundant literature showing that these problems are observed in many other sports [see (1, 2) for reviews]. For instance, the distribution of athletes' birthdate is still very biased in football (soccer), with an advantage for children born early in the year (16). The problem also occurs around the world in sports like weightlifting (17) and karate or fencing [de (18)] that are not as popular as football. The problem is also prevalent in female sport (19), and even extends to the risk of developing sedentary behaviors, with relatively younger people being less likely to engage in physical activity (20).

Since early born players (e.g., first 6 month of a birth year) are most likely to persist in competitive sport (1, 2) or, at least, to receive priority in terms of early selections, it is possible they are more likely to develop the interest and expertise necessary to become a coach. While becoming a coach does not necessarily require having been a good or successful player, based on the differences in early engagement in sport between relatively older and younger youth, and the relationship between feelings of competence and enjoyment with sustained engagement over time, it is reasonable to posit the hypothesis that there might be a birthdate effect among ice hockey coaches. This hypothesis is even more plausible considering that a study by Cobley and colleagues (21) on the history of elite soccer in Germany (in the Bundesliga) revealed a birthdate effect applying to elite coaches but not to referees. Similarly, Schorer et al. (22) revealed an overall birthdate effect amongst leadership (i.e., coaches, referees, and commissioners) in elite basketball.

The outcomes of relative age effects are well known, but we know considerably less about these effects on the likelihood of becoming a coach. Therefore, the purpose of this study was to examine whether the birthdate distribution in a large sample of ice hockey coaches reflected the bias observed among players. Considering the previous German studies in elite sport, we posited the hypothesis that there would be a birthdate effect on chances to become a coach. This hypothesis will be tested in the present investigation on a large pool of coaches in a sport (ice hockey) and province (Québec) where the birthdate effect amongst players in known to be strong (5).

## Methods

### Participants

The study population consisted of coaches involved in Québec minor ice hockey leagues (i.e., coaching players being 5–21 years old). All coaches in the sample (*n* = 3,380) were registered to Hockey Québec for the 2023–2024 season. Hockey Québec is the main organization dedicated to the development of ice hockey in Quebec and brings together approximately 85,500 players and more than 18,500 coaches.

A dataset was prepared and provided as an Excel file by the Program and Training Director of Hockey Québec. The file contained information about coaches’ birthdate, town of residence, and gender. The file was verified by the first author to make sure there was multiple entries about a coach.

Birthdates of 3,380 coaches were analyzed. Participants were born between 1952 and the end of 1979 (n = 949), between 1980 and 1989 (*n* = 1,855) or from 1990 to 2009 (n = 576).

This study was approved by the *Comité d'éthique et de la recherche de l'Université Laval*.

### Material and procedure

The Excel file with the birthdate of coaches was sent by the Program and Training Director of Hockey Québec to the first author. This information was then classified as a function of birth trimester and birth semester, and as a function of the year of birth.

Because the majority of coaches were born in the 1980s, we divided the sample into three periods (1952–1979; 1980–1989; 1990–2009), each with a large sample. People born in the 1980s were about 33–43 years old at the beginning the 2023–2024 season; it is reasonable to assume that in this age range, coaches have a child in age to be registered in a minor hockey league.

The coach data were contrasted with the distribution of births, by trimester and by semester, in the general Québec population for the same year-of-birth periods. Note that particularly for the 1990–2009 group, dividing into trimesters (Jan–Mar; Apr–June; July-Sept; Oct–Dec) and semesters (Jan–June; July–Dec) comes with an artifact that cannot be controlled. Some of these coaches may have played a part of their minor hockey between 2002 and 2008. During these years, the cutoff date for assigning players into categories was not January 1st, but October 1st. This artifact probably also touches some of the coaches born at the end of the 1980s.

Birth data since 1995 from the general Québec population were available on the internet [*Institut de la statistique du Québec* website (23)]. Birth data from 1975 to 1994 were provided by the *Institut de la Statistique du Québec* (data sent to the first author). Data from 1958 to 1972 were drawn from Grondin (24). The mean distribution of births from 1980 to 1989 and from 1990 to 2009 served to establish the expected distribution of coaches' births for these periods. For the 1952 to the 1979 period, the expected value was based on the mean for years 1958–1972 and 1975–1979.

The distribution of births in the general population in the 1990–2009 group was: 24.09% for Trimester 1 (January–March), 25.99% for Trimester 2 (April–June), 26.23% for Trimester 3 (July–September), and 23.68% for Trimester 4 (October–December). For the 1980–1989 group, the distribution was (in the same order): 24.31%, 26.18%, 25.71%, and 23.80%. For the other group (1979 and less), the distribution for Trimesters 1–4 was: 24.60%, 26.68%, 25.55%, and 23.17%.

## Results

The distribution of birthdates of coaches is presented as a function of three periods: born before 1980, in the 1980s, and after 1989. This distribution is presented per trimester and per semester in Table 1.

**Table 1:** Distribution of birthdates per trimester and per semester of coaches born before 1980, in the 1980s, and after 1989.

Trimester	Before 1980	%	1980s	%	After 1989	%
Jan–Mar	222	23.39	465	25.07	155	26.91
Apr–Jun	245	25.82	526	28.36	166	28.82
Jul–Sept	233	24.55	451	24.31	130	22.57
Oct–Dec	249	26.24	413	22.26	125	21.70
Semester		%		%		%
Jan–Jun	467	49.21	991	53.42	321	55.73
Jul–Dec	482	50.79	864	46.58	255	44.27
Total	949		1,855		576	

**Figure 1:**
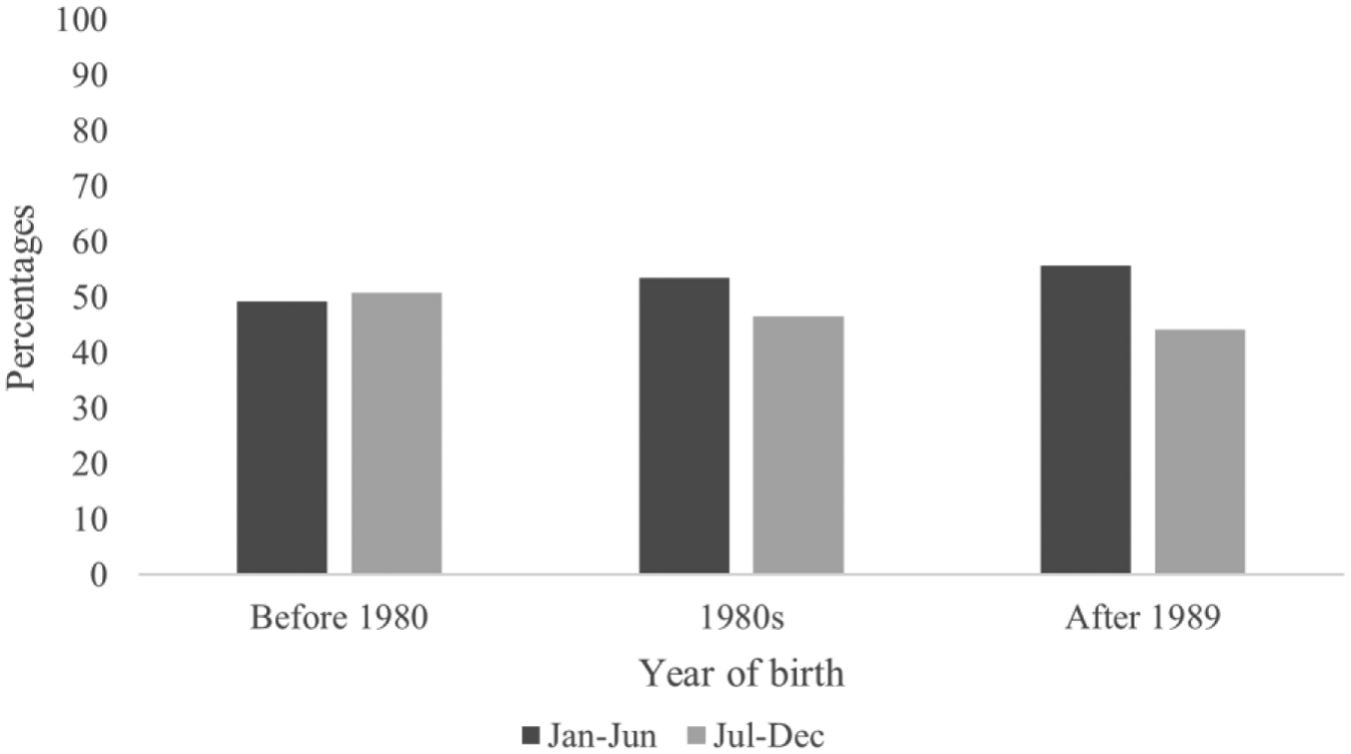
Percentages of births of coaches per semester in each group.

The distribution of coaches' birthdates was contrasted with the distribution of births in the general Québec population using chi square analyses. For the group of younger coaches (born since 1990), the analysis by trimester did not reveal any significant differences, *χ*²(3) = 7.56, *p* = .06. However, when the data was analyzed by semester, there were significantly more coaches born in the first semester than in the second, *χ*²(1) = 7.33, *p* = .006. Similarly, for coaches born from 1980 to 1989, the analysis revealed no differences in the distributions by trimester, *χ*²(3) = 7.04, *p* = .07, but a significant effect when examined by semester, *χ*²(1) = 6.38, *p* = .01. For the group of older coaches (born before 1980), the distributions differ neither when analyzed by trimester, *χ*²(3) = 5.06, *p* = .17, nor by semester, *χ*²(1) = 1.62, *p* = .20 (see Figure 1).

## Discussion

The objective of the study was to determine if there is a birthdate effect amongst coaches in minor league hockey. The data indicated a significant birthdate effect amongst coaches in the two younger groups of coaches. Those born in the first semester of the year were more likely to become coaches in youth ice hockey than coaches born in the second semester of the year. To our knowledge, there is no explanation for this biased distribution of births amongst coaches other than as a consequence of the birthdate effect amongst players. It is possible that many individuals who become coaches were former players. Because older players in a given cohort are more likely to access higher participation and competition levels in ice hockey, they are more likely to develop expertise in the field and an attachment with the discipline. This could positively influence the motivation to pursue involvement in ice hockey after their career as an active player. Alternately, younger players of a cohort are less likely to have success and to reach higher levels and are, therefore, less likely to develop the expertise in the discipline necessary to become a coach. In other words, the consequences of the relative age effect in youth sports go beyond player development; they affect chances to contribute to that sport later as an adult.

Knowing that there are more coaches born in the first half of the year, it is less surprising to observe that there are more coaches at higher levels born earlier in the competition year in sports like soccer (21). As well, if a longer participation in a sport provides expertise, attachment or identification to that sport, with the motivation to contribute somehow, it is not surprising to see a birthdate effect amongst other contributors to the game at higher levels, like referees or umpires (22).

In the groups of youngest coaches (born after 1989), the difference between Semester 1 and Semester 2 was quite large (11.5%). A closer look at the data indicates that the difference between Trimester 3 and Trimester 4 for this group was quite small (less than 1%) considering the distribution of births in the to population for these trimesters. This small difference could be due, as indicated in the Methods, a small artefact in our sample, and it was not possible to correct it in our dataset. Assuming that the birthdate effect in coaches is due to the relative age effect as a player, it becomes important to note that, in Québec, from the hockey season 2002–2003 to season 2007–2008, the cutoff date for categories was not January 1st, but October 1st (note that cutoff date for school in Québec is Oct 1st and that it was changed back to Jan 1st in hockey since 2008). This change in the cutoff date rapidly moved back the age advantage [Trimester 4 became Trimester 1; see (25)]. Therefore, a part of players born in the 1990s may have had a portion of their curriculum where the October–December trimester was not the fourth of the competition cohort, but the first. It was not possible to quantify the effect of this on the persistence of players and on their eventual chances to become coach, but it helps to understand why there was not much difference in the representation of coaches in Trimester 3 (4 during a brief period) and 4 (1 during a brief period), although there are much more births in the Québec population after 1989 in the Jul–Aug period than during the September–December period.

Interestingly, the birthdate effect was absent amongst coaches born before 1980. This effect would make sense if the birthdate effect on players was less pronounced for people born before 1980; however, this hypothesis can be rejected considering old data on the relative age effect in ice hockey. In Grondin et al. (5), for the 1981–1982 season in Québec, there was already a very striking effect in youth players, in other words for players born in the 1960s and 1970s [see (26)]. In addition, there was already a relative age effect in the NHL for that season (5). Around the same period, Barnsley et al. (4) also observed the same phenomenon amongst minor league players in Western Canada.

It is possible that the fading of the birthdate effect for the older group of coaches is connected to, or indicative of, the “reversal effect” seen amongst players. This effect is related to the decline of the strength of the birthdate effect as players get older, that is reaching higher levels of competition like the NHL in the case of ice hockey (27, 28). The reversal effect refers to the tendency to have more players who, when developing in minor hockey, were among the youngest in their age group than players who were among the oldest. This reversal effect has also been observed in elite football (soccer) leagues, in taekwondo (29), and in U17 football in the case of athletes from Africa (30). However, this reversal in player samples is often related to declines in the age advantage over the years; there could be no such direct effect amongst coaches. It may be that the youngest (disadvantaged by the birthdate effect) players who have been able to persevere in the practice of ice hockey do so because of skills [e.g., self-regulation; (31)] that relate to becoming a good coach. This idea is consistent with Andronikos et al. (29) who argued that a long-term benefit of being amongst the younger athletes in an age group is the necessity to develop early the toolbox of essential skills, skills that will eventually be necessary for competing at senior elite levels. For instance, key self-regulation skills are planning and reflection, which would obviously be useful tools for developing coaching capabilities. This might explain why they are less likely to quit coaching as they get older. While this hypothesis is highly speculative, our results suggest the influence of birthdate (and its reversal) extends beyond an athlete's playing career.

Despite the intriguing results of the current study, our investigation had some limitations. One limitation, noted above, is related to the changes in the cutoff birthdate used for age grouping where our sample was drawn (Québec). It is possible that what is called Trimester 4 in our analysis may have also been, for a brief period, Trimester 1 for some coaches when they were playing. This certainly induces a bias in our analyses, and indeed if we look at the number of coaches born in Trimesters 3 and 4 in the second half of the 1980s (1985–1989; i.e., for a segment where coaches may have had a part of their years as a player under the Oct–Dec trimester as Trimester 1), the distribution is approximatively equal. When looking at the first half of the 1980s, there were about 53% and 47% of coaches born in Trimester 3 and 4, respectively. Note that the potential consequence of this bias is that it probably reduces the magnitude of the birthdate effect observed with coaches.

Although not, strictly speaking, a limitation of the study, the nature of the sample, should be kept in mind when reading the study. There were many more coaches born in the 1980s than in the 1990s or 1970s. As indicated in the Methods, at the time of the study (the 2023–2024 season), coaches born in the 1980s were between 33 and 43 years old, ages where people often have their own children, old enough to play minor league hockey. It is reasonable to posit that a good proportion of these parents had children on the team (32). Once their child quit organised hockey, the parent of the child often leaves as well. Only people having real aptitude for coaching, having for instance more planning and reflection skills, will keep being involved in ice hockey. This would explain why the relative age effect fades in the sample of older coaches: there is no reason to believe that planning and reflection skills are related to birthdate.

The first practical implication of this finding is that grouping young athletes into categories based on chronological age not only provokes a potential loss of talented players, it may also eventually lead to a loss of potential coaches. A second implication is the tendency observed in the data to have a reversal effect with older coaches, which may reflect the need for the younger athletes in an age group to develop more rapidly essentials skills (29), including psychological skills and reflection (31).

The findings of the present study suggest several areas for future investigations. Because ice hockey is so popular in Canada, a first step would be to extend the investigation to other Canadian populations where the cutoff date has never been changed. This would not only clarify the magnitude of the birthdate effect in coaches, but also its potential reversal with older coaches. Such investigations about ice hockey could also be conducted in other countries where the birthdate effect, for ice hockey, was larger or smaller than in Québec [e.g., Russia; (13)], to see if the effect amongst coaches is modulated by these fluctuations, and to see if there is any such effect in countries adopting different criteria to become a coach. And of course, the investigation relative to the month, trimester or semester of birth of coaches must be extended to other sports, such as soccer and rugby where strong birthdate effects have been regularly reported (1, 2). In the context of the shortage of coaches in many sports, it is important to explore and understand the administrative and psychological elements underpinning this phenomenon.

## Data Availability

Publicly available datasets were analyzed in this study. This data can be found here: https://osf.io/64kt5/?view_only=573da4101af14ac58fcee3363024130fOpen Science Framework.
